# *Phosphenes* and the effects of charged particles on the visual system

**DOI:** 10.3389/fneur.2025.1621659

**Published:** 2025-09-16

**Authors:** Simone Carozzo, Walter G. Sannita

**Affiliations:** Department of Neuroscience, Neurorehabilitation, Ophthalmology, Genetics, Mother/Child Sciences (DINOGMI), University of Genova, Genova, Italy

**Keywords:** visual positive phenomena, space travels, hadrontherapies, mechanisms of generation, retinal activation, safety, phosphenes, electrophysiology

## Abstract

**Objective:**

Review the evidence from space travel crews, early and recent experiments in particle accelerators, and observations in patients undergoing heavy ions treatments for tumors of the eye or skull that charged particles act on the visual system triggering the perception of *phosphenes*.

**Results:**

The visual effects of charged particles are multi-modal in origin, act differently on the retina photoreceptors and neurons, and indicate a peculiar sensitivity to charged particles of the visual system. Acute toxicology in heavy ion treatment has been documented and should be differentiated by quasi-physiological actions.

**Significance:**

The potential relevance of *phosphenes* as indicative of functional impairment stands as a possible variable in hadron-therapy, the proper monitoring of which may contribute to optimizing the treatment procedures and in the prediction of outcome.

## Highlights

- Simple unstructured *phosphenes* first became a safety issue in space travels and then a common observation in hadron therapies.
- The mechanisms of generation remain largely unknown to date. Their reports by astronauts or irradiated patients are often overlooked, possibly due to the peculiar and extreme conditions in space travel and the low priority in the case of therapies with limited or null alternatives.
- Quantitative neurophysiological methodologies may help optimize the radiation treatment procedures and reduce the patients’ discomfort - and improve the understanding of the treatment mechanisms.

## 1 Introduction

Charged particles can act on and interfere with the mechanisms involved in visual information processing. Evidence in this respect comes from early reports by space crew members and later by patients undergoing heavy ions treatment of tumors of the eye or skull who reported *phosphenes*, i.e., unstructured positive visual phenomena (light flashes) occurring in isolation and in the absence of alterations of consciousness. Structured or complex positive phenomena (*photopsias, kinetopsia, visual distortions* or *hallucinations*) have not been described. Patients undergoing proton radiotherapy occasionally reported non-visual sensory percepts, often associated with *phosphenes* and with the brain regions receiving the highest doses corresponding to the anatomical structures associated with each type of percept (1–4). Non-visual percepts, either isolated or concomitant to *phosphenes* in multisensory perception, appear less frequent or exceptional in space (2, 3, 5–7). The origin of *phosphenes* in radiation activating structures of the retina or the optic nerve and some peculiar sensitivity of the visual system to charged particles have been hypothesized. Comparable percepts were reproduced in early human experiments in accelerator (8–14). The mechanisms of generation are conceivably complex and remain to be investigated in full detail. Occurrence, potential interference with CNS functions, and clinical relevance with respect to the pathophysiological conditions in which *phosphenes* are observable remain underestimated.

## 2 *Phosphenes* in disordered visual processing

*Phosphenes* are uncommon but can occur spontaneously also in a variety of physiological conditions such as transient deformation of the eye, accommodation, saccades, vivid lights, rhythmic light stimulation, etc. (15). The mechanical deformation of the eyeball, for instance, causes *phosphenes* by activating both center-on and center-off retinal ganglion cells (16). Excitation or the damage or functional impairment at any portion of the visual system (retina, afferent pathways or cortex) can result in positive visual phenomena, including *phosphenes*. These are reported in severe visual loss and by subjects using neuroactive drugs (mostly alcohol and hallucinogens), and occasionally in other disorders of the CNS (15, 17). The incidence of simple *phosphenes* in epilepsy is low (<1%) and limited to the focal involvement of visual cortex. They are by contrast quite common in the *aura* preceding pain in migraine attacks. Most studies agree in estimating the occurrence of *phosphenes* in a portion up to ~90% of adult *migraneurs*, irrespective of their experiencing visual auras with or without migraine. Functional testing of the visual cortex by transient magnetic stimulation (TMS) has documented a neuronal hyperexcitability in migraine with *aura* compared to both *migraineurs* without it and controls (18–23). In contrast, the threshold of TMS-induced *phosphenes* did not differ in a study comparing migraine without aura, migraine with aura, and control groups (68 ± 9.5% vs. 75 ± 12%, vs. 80 ± 11%, respectively) (24). The visual aura in migraines is associated with reduced cerebral blood flow and increased BOLD signals (25–27). Migraine, migraine aura without pain and headache are reportedly common during space traveling (with unclear incidence) and have been mimicked in studies on Earth (28, 29).

Colors and the perception of movement can result from the stimulation of basal occipital areas in the fusiform and lingual gyri and of the parieto-occipital and basal temporo-occipital junctions, respectively. Activation/damage of specific portions of the visual system has been correlated to structured or complex positive visual phenomena (hallucination, etc.), but no topographic selectivity (or significantly higher incidence) has been found thus far for simple percepts like *phosphenes* (15, 17, 30, 31). Bilateral cortical activation covering the entire visual cortices was observed by fMRI in patients with Leber’s hereditary neuropathy of optic nerve reporting induced *phosphenes* (32). *Phosphenes* have been reported during hypothalamic deep brain stimulation (33).

## 3 Particle-related phosphenes

### 3.1 Phosphenes in space missions

Crewmembers on Apollo, Skylab, MIR and International Space Station (ISS) missions reported *phosphenes* occurring spontaneously in the form of unstructured light flashes. Reports appear to have been quite common since the first years of space exploration, according to early NASA reports. About 80% of astronauts reported having perceived *phosphenes* at least in few missions and often over several orbits, according to a retrospective survey (5). Detailed reports have described flashes, sparkles, zigzag lines, supernova or the like, either static or apparently moving across the visual field or toward the observer (vertical motion does not seem to have been observed) (5, 6). Colors (e.g., yellowish, pale green or blue) were described only occasionally. Although with individual differences, *phosphenes* occurred at average rates that varied depending on the spacecraft shielding, orbital height and latitude and were correlated to the cosmic radiation flux, which in orbit is modulated by the Earth magnetic field. Their rate varied between ~0.02 and ~16 per minute, it was higher near the magnetic poles than in equatorial latitudes and highest during the passages across the South Atlantic Anomaly over Brazil where the magnetic field is lower in altitude. About 1.3 *phosphenes* per minute on average were reported outside the geomagnetic shield during the transit to the Moon (2, 6, 8, 9, 13, 34, 35). A temporal correlation between *phosphenes* and particles flux also providing information about the particle trajectory and charge was detected by particle detectors onboard the MIR station (13, 35) and in the International Space Station (2).

### 3.2 Phosphenes in heavy ions therapies

*Phosphenes* have been observed during early exposure to X-ray and then in patients undergoing carbon ion or proton therapies of tumors of the eye or head (1, 2, 14, 36–39), with reported incidence increasing with the interest on the possible mechanisms of generation. They appear common and are described by over 2/3 of subjects according to retrospective surveys and prospective studies. White light flashes are reportedly more common in carbon ion treatment, but blue/violet percepts proved the more frequent across all treatments with colors also depending on light/dark adaptation and on the distance of local irradiation from the fovea and the predominant stimulation of rods or cones (4, 36, 39). *Phosphenes* are usually reported by patients as mildly unpleasant; dose, age, history of food allergies or longer duration of disease can predict higher intensity of perception (4, 40). A retrospective study of patients undergoing head or neck proton radiotherapy indicates that small numbers of protons can evoke neuronal responses on a 0.1 s time scale able to startle conscious percepts comparable to those from normal sensory inputs; the regions of the brain receiving the highest doses reportedly correspond to the anatomical structures associated with each type of percept (14, 39). The relationship of *phosphenes* incidence with the accelerator beam positions and the tumor distance from the fovea and the optic disk in patients with skull base tumors suggest an origin with irradiation of the retina or anterior portion of the optic nerve (14, 37, 38) that may deserve comparison with the effects of direct electrical stimulation of the optic nerve or visual cortex. Monitoring by standard EEG and retinal and cortical electrophysiology at the end of a full session of ^12^C treatments of skull chordomas and chondrosarcomas unsuitable for radical surgery described both improvement due to reduction of the mass tumor in most patients and acute functional impairment of small severity and possibly transient in about 1/3 of subjects (41). The correlation between these electrophysiological indicators of functional impairment and the incidence of *phosphenes* has not been investigated, nor has the possible relevance as potential markers of retinal or brain damage in the prediction of outcome (42). Abnormal olfactory sensations (*phantosmias*), either isolated or most often accompanied by *phosphenes*, proved relatively frequent in a prospective study; the association of these percepts has been proposed as due to direct/indirect irradiation of brain structures.

### 3.3 Studies in particle accelerator

#### 3.3.1 Early human experiments

*Phosphenes* comparable to those described by astronauts were reproduced under controlled experimental conditions in particle accelerators in the early 70s’ (5, 37). The eyes of healthy volunteers (usually the scientists themselves) were exposed to single particles or collimated particle bursts in the hundred MeV energy domain. Bursts of few relativistic muons, pions, neutrons, nonrelativistic helium, nitrogen, carbon ions, etc. were tentatively applied to identify the differences between and the possible relevance of the particle physical properties. Minimally ionizing particles emitting Cerenkov radiation (i.e., the effect of charged particles traveling through a medium at speed higher than the light in the same medium) produced visible light and the volunteers reported large *phosphenes* as predictable. Discrete *phosphenes* were nevertheless reported also with highly ionizing particles (e.g., HZE nuclei) at energies *below* the threshold producing Cerenkov visible light, but always and only upon passage through the posterior portions of the eye. *Phosphenes* were described as short in duration, without after-image, with approximate correlation between the irradiated retina and the portion of visual field in which *phosphenes* were subjectively located. Motion in the same direction of the beam was often reported (10–12, 43–45). The perception of *phosphenes* following exposure to accelerated nitrogen nuclei below Cerenkov threshold had an estimated efficiency between 10 and 40%, with the differences among subjects and across studies being possibly accounted for by the experimental conditions, number of particles, etc. (8, 10, 11). Indirect Cerenkov visible radiation can be produced also by ions below threshold via a direct Cerenkov emission (1, 46) as *phosphenes* of diffuse bluish light.

#### 3.3.2 Electrophysiological experiments in rodents

The eyes of anesthetized wild-type mice were rhythmically irradiated with short (~5 ms) bursts of ^12^C ions under control conditions in particle accelerator, with the beam collimated in diameter and directed approximately perpendicular to the eye posterior pole. ^12^C ions evoked electrophysiological retinal mass responses comparable to those generated by light and initiated retinal events yielding to a cortical response, with increased latencies and decreased amplitudes of both responses. The results appeared compatible with mechanisms of ^12^C action mimicking the light in triggering the retinal photoreceptors. However, the retinal response amplitude increased linearly to a maximum at ~2000 ions/burst (0.72 mGy/burst) intensity to then decline at higher numbers of ions/burst in an inverted-U relationship (47). The association of ^12^C bursts with light stimuli (white flashes) in the same animal and sequence of stimuli reduced the amplitude of *waves a* and *b* of the mass *electroretinogram* and increased the amplitude and *phase-locking* to stimulus of the (post-synaptic) oscillatory retinal responses and of cortical responses (48). These findings collectively suggest a complex action of ^12^C ions on (and functional interference with) retinal structures/mechanisms resulting in cortical activation that exceeds a direct action of photoreceptors (47, 48).

## 4 Particle-related positive visual phenomena and CNS functions

Retinal photoreceptors and neurons in the retina and cortex are sensitive to ionizing agents and can respond to their action with transient functional changes. *Phosphenes* have been reported by subjects exposed to X-rays, with the *electroretinogram* amplitude correlating with the X-rays intensity; an action on the rhodopsine proteic component opsin starting a cascade of reactions leading to a visual sensation has been suggested (49, 50). Focal electrical stimulation of striate visual cortices V_1_ and V_2_ during neurosurgery evoked simple *phosphenes*, while stimulation of the extrastriate areas or temporal regions evoked complex visual phenomena (15, 51, 52). In early studies, electrical stimulation of discrete points of the human visual cortex produced corresponding punctuated sensation of light in both sighted and blind subjects. Systematic investigation on the size, luminosity and position in visual space of *phosphenes* induced by discrete electrical stimulation of the retina and optic nerve has been instrumental in the tentative development of prostheses for the blind; electric stimulation is thought to evoke *phosphenes* by opening voltage-sensitive ion channels and by-passing the chemically gated channels in stimulated neurons (53–58). Magnetic stimulation of the retina or cortex induces *phosphenes* via complex mechanisms of action and depending on the stimulate position and functional status (59–62).

A suggested alternative mechanism of generation is a local disinhibition of neuronal structures which can result in increased neuronal excitability due to deafferentation (the *release* phenomenon). In this regard, any local cause of deafferentation within the visual system able to interfere with information processing via functional impairment (e.g., miscoded neuronal function) or due to anatomic damage (macular degeneration, glaucoma, cataract, macular holes and other retinopathies, lesions of visual pathways or cortices) can result in a release mechanism and in the perception of positive visual phenomena (14, 63). Retinal or cortical damage has not been observed in *in vivo* rodent studies with direct retinal stimulation below the Cerenkov threshold (47). However, detrimental effects on synaptic density and myelination in response to exposure to high-energy charged particles have been reported (64). Significant retinal damage (apoptosis in vascular endothelial cells, significant changes in regulated protein expression, cellular structure, immune response and metabolic function) have been observed in mice after 35 days in space and were reduced by artificial gravity (65).

The findings collectively suggest some action of heavy ions on photoreceptors and a parallel activation of postsynaptic currents in inner retina, with modalities not necessarily comparable to those of light, but nevertheless able to trigger the retinal cascade to cortical activation (48). Studies on patients with retinitis pigmentosa and mutant mice have documented activation of the visual cortex in response to light also after photoreceptors extensive damage (49, 66–68). Subsets of intrinsically photosensitive retinal ganglion cells have been identified in animals. In primates, these cells project to the lateral geniculate nucleus and reportedly merge with the retinal pathways to cortex that transfer visual information to be processed into visual images (69–71); some role in the generation of *phosphenes* has been suggested (38). Transient depression of neuronal activity (often referred to as *spreading depression* in rodent models) can be associated to increased released of K^+^, propagation of Ca^2+^ between glia and neuronal cells or glutamate action (15, 72) and has been observed in cortex, hippocampus, and in superior colliculus and retina.

Evidence suggests that photon emission can mediate in networks function as an additional signal enabling cell-to-cell communication and coupling depending on the neuronal physiological state (73). Bovine rhodopsin irradiated with ^12^C ions in suspension was activated by ions as it was by light. The process was mediated by lipid peroxidation and successive chemiluminescence and the bleaching process proved reversible under these experimental conditions. These findings indicate a ^12^C ions-induced recombination of radicals possibly responsible for the release of photons with subsequent bleaching of rhodopsin and the triggering of mechanisms generating *phosphenes* (74, 75).

## 5 Comments

Different mechanisms are suggested to mediate in the production of *phosphenes* and are individually or collectively eligible as generating processes (38). However, the interest on the issue has encompassed different scientific contexts, with varying technological background, rationale, and experimental approaches. Research on the topic has been fragmentary rather than systematic, and the available evidence may suggest limited depth of investigation. The different radiation and conditions of irradiation causing *phosphenes* propose the transfer of energy as a conceivable common mechanism activating photoreceptors and/or neuronal cells and their interaction in the retinal cascade. Interactions between the charged particles action and the retina and/or the CNS functional status of the stimulated cells or network are also conceivable. The Cerenkov radiation measured in particle accelerator experiments was compatible with the estimated threshold sensitivity to photons of the retina photoreceptors, with mechanisms of generation mimicking light (10, 12). Indirect Cerenkov light due to nuclear interactions/radioactive decay or direct activation/radical excess within the retina are also possible. Models and experiments nevertheless estimated a threshold number of ionizations per sensitive volume below the Cerenkov threshold, indicating effects on the rod outer segment or photochemical molecules at the energies compatible with those used for ocular proton therapy (11) or able to activate network functions (1, 2, 42, 63). A direct action of ionizing particles on photoreceptors or neural tissues in the retina appears compelling but is not conclusive. Direct effect on brain neurons remains undocumented and *in vivo* and *in vitro* experiments implicate mechanisms of action on the visual system that could be more complex than hypothesized and depend to a relevant extent on the activated cells or structures. The bilateral cortical activation covering the entire visual cortices observed by fMRI when patients reported *phosphenes* for instance indicates a relevant impact on visual function mediated by magnification factors (16, 32). Several subjective characteristics of the *phosphenes* reported in space, in *in vivo/in vitro* experiments, and during hadron therapy are similar, but a scale problem result of the wide range of the ions number needed to generate a subjective *phosphene* or obtain a chemical or electrophysiological response. Small numbers of particles may elicit a subjective *phosphene* in space, although not every single ion can, with and estimated efficiency in the order of 10^−2^–10^−3^. The threshold *in vivo* animal models is ~10^3^ ions; higher flux appears necessary both in *in vitro* models and in patients undergoing hadron therapies (2). The functional status of the stimulated areas and network are also conceivable. For instance, about 25% of subjects do not report perceiving *phosphenes* upon magnetic occipital stimulation (76). The estimated radiation release in hadron therapy accounts for both normal tissues and target tumors, so that single pulse delivery or full irradiation are to be considered in *in vivo* and *in vitro* studies, respectively.

Evidence from laboratory tests suggests that the *phosphenes* reported by astronauts may reflect transient functional impairment. Their relevance as potential early markers of later biological tissue damage remain largely unknown and would add health hazard to the effects of microgravity, isolation, disruption of circadian rhythm, impaired sleep dynamics, and hypercapnia associated with space travel [e.g., (77)]. Long-duration space travels nevertheless impose conditions that are known to interfere with and may have detrimental effects on the visual system and CNS functions in humans also irrespective of heavy ions exposure (7, 78–82). The limited number of particles triggering phosphenes in space may indicate a limited risk or reinforce the hypothesis of higher sensitivity of the visual system to radiation direct/indirect action due to the prolonged permanence in microgravity (73), and combined effects of particles and microgravity in space appear therefore peculiarly probable. Strategies to counterbalance the effects of microgravity have been suggested and methods to shield astronauts from cosmic radiation have been devised and are being tested (7, 83–87). On the contrary, the effects of therapeutic irradiation on the photoreceptors and nervous system and the mechanisms originating *phosphenes* in patients have been largely overlooked thus far, most likely because of the low priority of these phenomena in the estimated cost–benefit ratio of a therapeutic approach with limited or no alternatives. Acute side-effects on encephalic nervous tissues have been described in patients during and immediately after ^12^C treatments of skull tumors (41), but long-term monitoring is not yet available and the possible relevance of *phosphenes* as early markers of visual impairment has not been and should be investigated in full detail. The action of particles on the visual system may be deemed of limited relevance in the extreme conditions of space travel, but further research may devise application in therapeutic procedures that are becoming routinary and better tolerated by patients. The correlation of *phosphenes* and their conditions of occurrence in hadron therapies with quantitative neurophysiological measures may provide additional information on the pathophysiology of charged particles effects on CNS tissues. More significant could be this information in further improving the procedures of hadron treatments and the prediction of outcome, and in reducing the patients’ discomfort (88).
